# Surgical safety of cervical pedicle screw placement with computer navigation system

**DOI:** 10.1007/s10143-016-0757-0

**Published:** 2016-05-31

**Authors:** Nobuyuki Shimokawa, Toshihiro Takami

**Affiliations:** 1 Department of Neurosurgery, Tsukazaki Hospital, 68-1 Waku, Aboshi-ku, Himeji, Hyogo Japan; 2 Department of Neurosurgery, Osaka City University Graduate School of Medicine, Osaka, Japan

**Keywords:** Cervical spine, Image-guided surgery, Pedicle screw, Posterior cervical fixation, Spinal instrumentation

## Abstract

Cervical pedicle screw (CPS) may be the biomechanically best system for posterior cervical segmental fixation, but may carry a surgery-related risk. The purpose of this study was to evaluate the safety of CPS placement using computer navigation system for posterior cervical instrumented fixation and discuss its complication avoidance and management. Posterior cervical instrumented fixation using CPS was performed in a total of 128 patients during the period between 2007 and 2015. Intraoperative image guidance was achieved using a preoperative 3D CT-based or an intraoperative 3D CT-based navigation system. A total of 762 CPSs were placed in the spine level of C2 to Th3. The radiological accuracy of CPS placement was evaluated using postoperative CT. Accuracy of CPS placement using a preoperative 3D CT-based navigation system was 93.6 % (423 of 452 screws) in grade 0; the screw was completely contained in the pedicle, and accuracy of CPS placement using an intraoperative 3D CT-based navigation system was a little bit improved to 97.1 % (301 of 310 screws) in grade 0. CPS misplacement (more than half of screw) was 3.3 % (15 of 452 screws) using a preoperative 3D CT-based navigation system, and CPS misplacement (more than half of screw) was 0.6 % (2 of 310 screws) using an intraoperative 3D CT-based navigation system. In total, 38 screws (5.0 %) were found to perforate the cortex of pedicle, although any neural or vascular complications closely associated with CPS placement were not encountered. Twenty nine of 38 screws (76.3 %) were found to perforate laterally, and seven screws (18.4 %) were found to perforate medially. Image-guided CPS placement has been an important advancement to secure the safe surgery, although the use of CPS placement needs to be carefully determined based on the individual pathology.

## Introduction

Posterior cervical instrumented fixation has undergone revolutionary changes over the last several decades, since posterior cervical stabilization using lateral mass screw (LMS) was first described by Roy-Camille et al. in 1979 [1]. Various surgical techniques of interlaminar wiring, LMS, transarticular screw, and most recently, cervical pedicle screw (CPS), are available [2–9]. Successful clinical application of CPS for traumatic cervical injury was first reported by Abumi et al. [1], and posterior cervical instrumented fixation using CPS is gaining popularity and can be applied for cervical spine injury and for several cervical pathological conditions, such as cervical spondylotic myelopathy (CSM) with instability, kyphotic deformity, congenital anomaly, or neoplastic destruction. CPS may be the best biomechanical system for posterior segmental fixation [10–15], but may also carry a surgery-related risk of neural or vascular complications [3, 16–19]. On the other hand, the popularization of posterior cervical instrumented fixation has raised an important issue of safe surgical management. The purpose of this study was to evaluate the accuracy of CPS placement using computer navigation system for posterior cervical instrumented fixation and discuss its complication avoidance and management.

## Material and methods

### Patient population

Posterior cervical instrumented fixation of the occipitocervical, cervical, or cervicothoracic spine using CPS combined with/without LMS was performed in 128 patients at our institute between 2007 and 2015. The patients included 84 men and 44 women, and the mean age at surgery was 65.5 years (range, 15–92 years). The clinical diagnosis was CSM with instability in 50 patients, trauma in 35 patients, reducible cervical kyphosis in 13 patients, CSM associated with athetoid-type cerebral palsy in 11 patients, destructive spondyloarthropathy (DSA) in 5 patients, rheumatoid arthritis (RA) in 4 patients, congenital anomaly in 4 patients, neoplastic destruction in 4 patients, and infection in 2 patients. The patient characteristics are summarized in Table 1.

**Table 1:** Patient characteristics

Characteristics	No. (range)
Total no. of patients	128
Sex (male/female)	84/44
Mean age at surgery (years)	65.5 (15–92)
Postoperative follow-up (months)	51.8 (6–121)
Preoperative diagnosis	
CSM with instability	50
Trauma	35
Reducible cervical kyphosis	13
CSM associated with athetoid-type cerebral palsy	11
DSA	5
Anomaly	4
Tumor	4
RA	4
Infection	2

*CSM* cervical spondylotic myelopathy, *DSA* destructive spondyloarthropathy, *RA* rheumatoid arthritis

### Preoperative evaluation

**Fig. 1:**
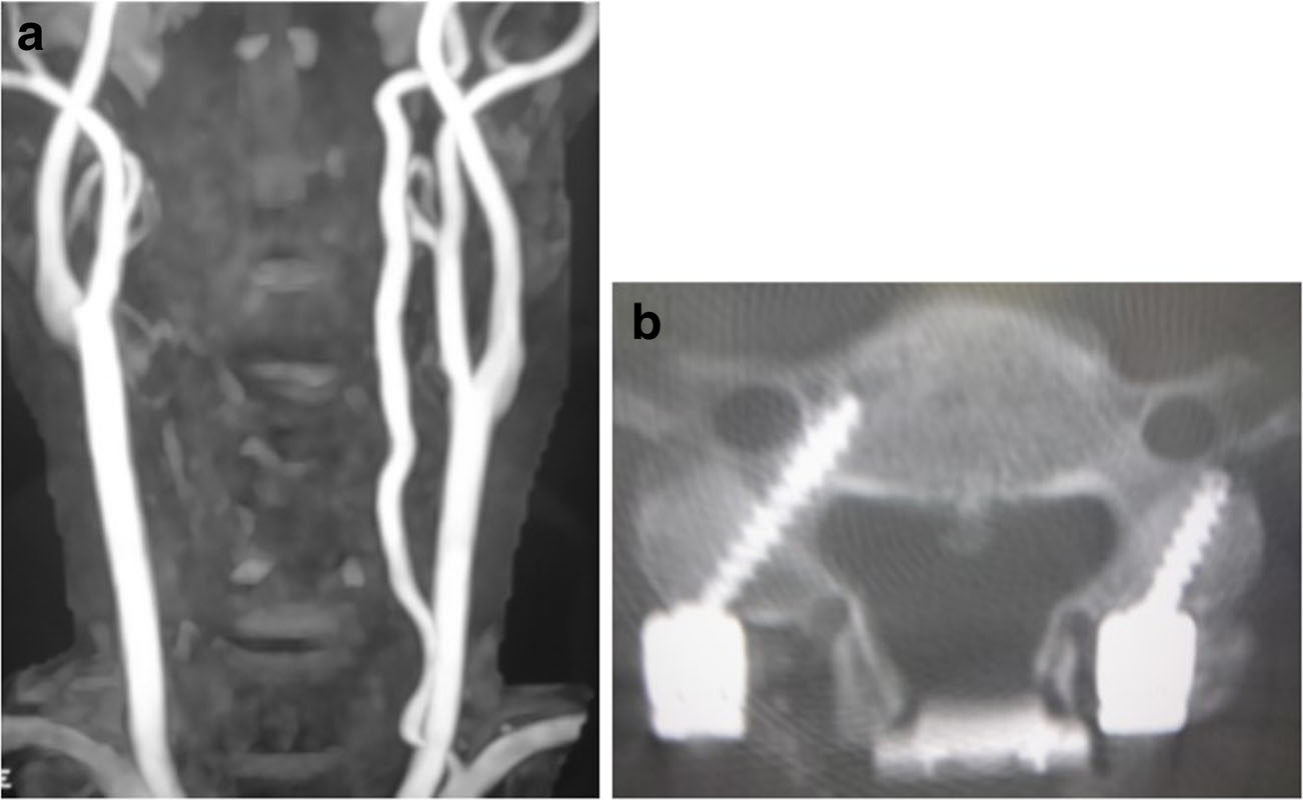
**a** Selection of cervical pedicle screw (CPS) and lateral mass screw (LMS) in cases in which preoperative MR angiography shows occlusion of the vertebral artery on the right side. **b** Postoperative CT shows that CPS was placed on the right side and the LMS on the left side

Comprehensive radiological evaluation was routinely accomplished. Pedicle morphology (coronal and axial, sagittal) and the medial inclination of the pedicle axis were carefully evaluated. CPS was not indicated is cases with a pedicle diameter less than 4 mm, as determined by preoperative 3D CT imaging. The development and running course of the vertebral artery (VA) in the transverse foramen at each vertebra, and the collateral vascular network (i.e., posterior communicating arteries) were carefully evaluated with MR angiography and/or CT angiography. When occlusion of the unilateral VA was recognized before surgery, CPS was avoided on the remaining side. In such cases, CPS in the VA occluded side and LMS in the remaining side were performed (Fig. 1a, b).

### Surgical procedure

CPSs were placed using a preoperative 3D CT-based navigation system (Stealth Station and Stealth Station TREON^TM^, Medtronic, Inc., Memphis, TN, USA) during the first study period (2007–2012) and using an intraoperative 3D CT-based navigation system (O-arm and Stealth Station 7, Medtronic, Inc., Memphis, TN, USA) during the second study period (2012–2015). After standard exposure of the posterior cervical spine, CPSs were basically placed before the decompressive procedure such as laminoplasty or laminectomy. In the early series of a preoperative 3D CT-based navigation system, navigation frame was fixed to the spinous process of the vertebra where CPSs were placed. The accuracy of navigation was carefully adjusted by using the method of point registration and surface matching (separate registration), especially in case of the unstable cervical spine. The CPS insertion point was prepared using a 2-mm diamond burr. The screw hole was then dug gently using a navigation straight probe. The ballpoint sounder probe was used to make sure that the cortex of pedicle was not violated, and finally, the CPS was inserted under the guidance of lateral fluoroscopy. Navigation accuracy was repeatedly confirmed by touching the anatomical landmark of the posterior cervical spine before another CPS insertion. Even in the latter series of an intraoperative 3D CT-based navigation system, the accuracy of navigation was repeatedly confirmed within two above-two below vertebral levels at one fixed registration frame. In case of long fixation, the range of navigation registration was divided into several segments of four or five vertebral levels.

**Fig. 2:**
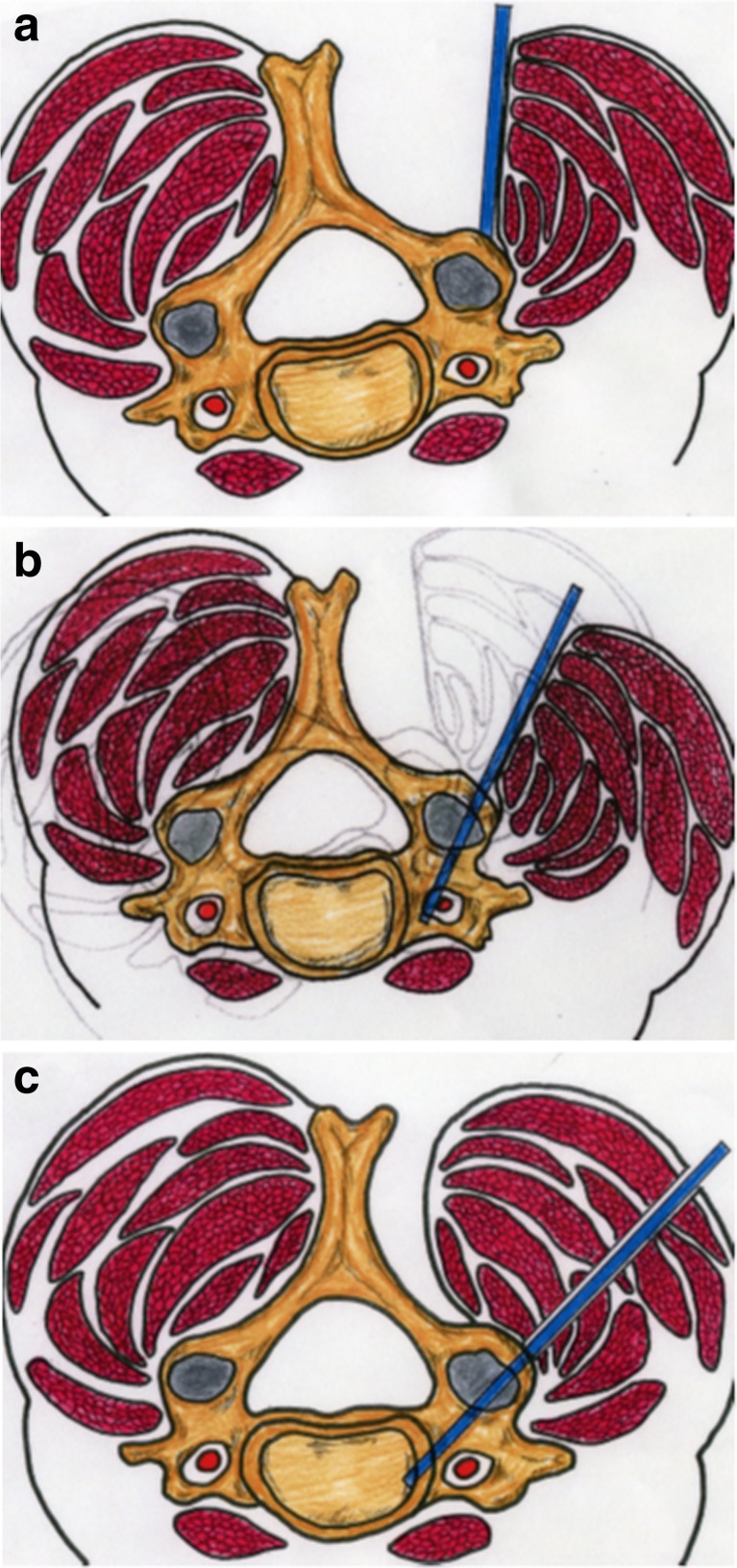
Illustrations showing that the axial trajectory of CPS is often restricted or pressed medially by the surrounding paravertebral muscles (**a**), and screws may be misplaced laterally (**b**). Percutaneous placement of CPS was a choice to resolve such a technical problem (**c**)

The axial trajectory of CPS is often restricted or pressed medially by the surrounding paravertebral muscles (Fig. 2a). The stronger the axial trajectory is kept against the medial pressure from the paravertebral muscles, the easier axial rotation or sagittal bending of the cervical spine might be, so that screws may be misplaced laterally (Fig. 2b). There might be a higher risk of VA injury. When extreme oblique trajectory of CPS through posterior midline approach was disturbed by the cervical paravertebral muscles, percutaneous placement of CPS was a choice to resolve such a technical problem (Fig. 2c).

After placement of CPSs, laminectomy or laminoplasty was performed if necessary. However, in cases of severe canal stenosis, such as combined or beak-type OPLL with instability, a decompression procedure was considered before CPS placement. CPSs were tightly connected using a rod system with/without spinal correction. In cases of neural foraminal stenosis, there is a risk of iatrogenic nerve root impingement due to worsening of foraminal stenosis that may be caused by a corrective reduction procedure. Excessive compression force at the segments was avoided in such cases. Finally, autologous local bone with beta tricalcium phosphate (TCP) was placed into the posterior surface of decorticated facet joints on both sides. After setting the screw-rod contouring, cervical alignment and correct position of all CPSs was confirmed using lateral fluoroscopy or intraoperative 3D CT with an O-arm.

### Statement of ethics

This is a retrospective case analysis. We certify that all applicable institutional and governmental regulations concerning the ethical use of clinical data were followed in the present study. Patient informed consent was obtained prior to surgery.

## Results

**Fig. 3:**
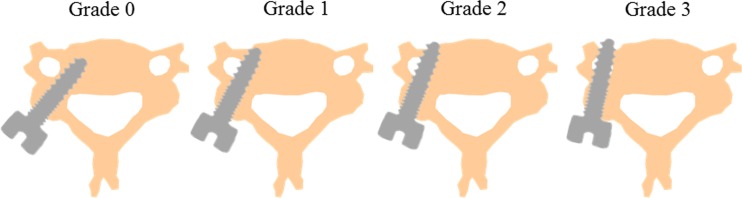
Illustrations showing the grading scale of the radiological accuracy of CPS placement evaluated using postoperative CT scan that was originally proposed by Neo et al. [14]

**Fig. 4:**
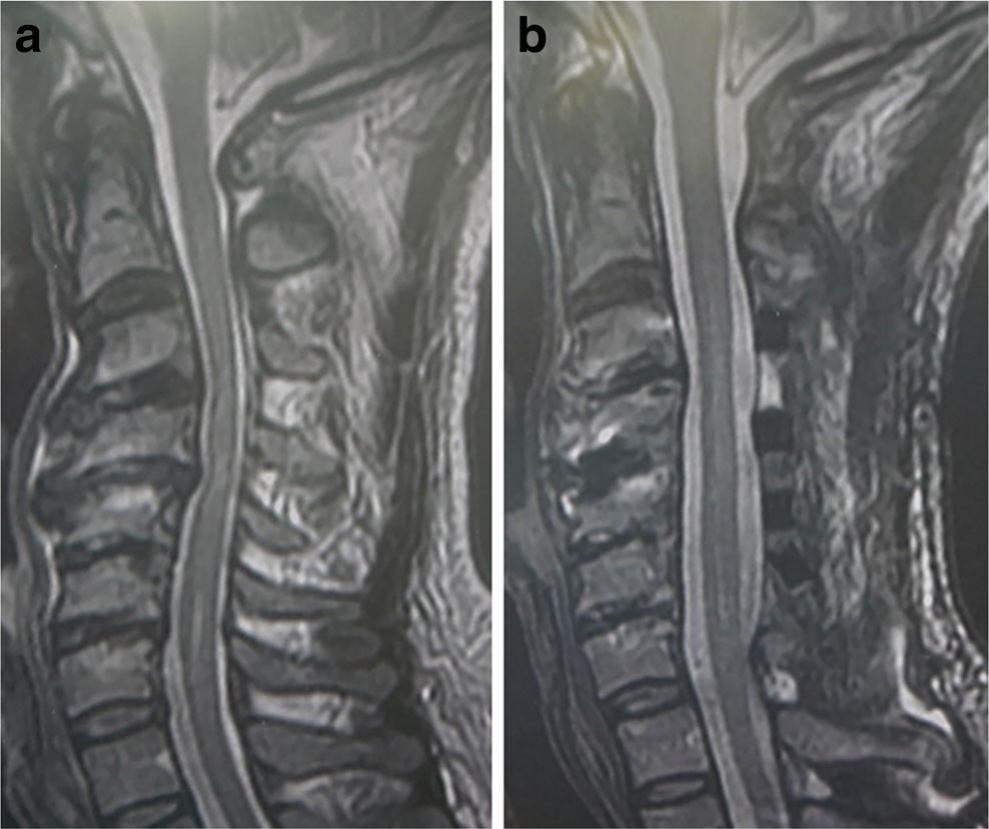
Illustrative case. A 47-year-old man presented with gradually progressive tetraparesis. He underwent posterior decompressive and corrective fixation using C2-T2 CPSs combined with anterior fusion. **a**. Preoperative MRI revealing cervical kyphosis resulting in cervical spondylotic myeopathy. **b**. Postoperative MRI

**Fig. 5:**
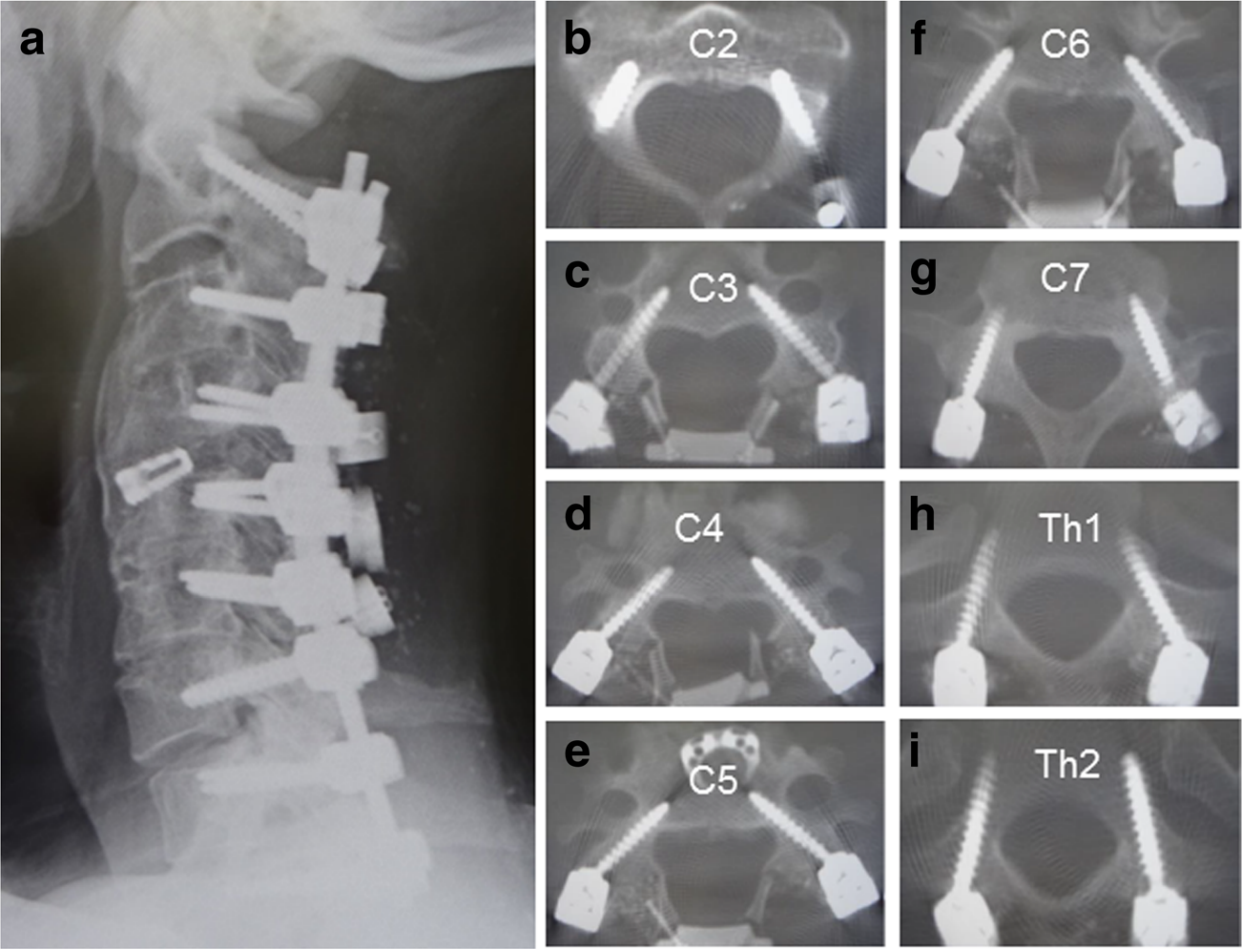
Illustrative case. The same patient in Fig. 4. **a** Postoperative plain cervical radiograph. **b**–**i** Postoperative axial CT scans at spine level from C2 to Th2

The radiological accuracy of CPS placement was evaluated using postoperative CT scans and was defined retrospectively by a grading scale originally proposed by Neo et al. [20]. (Fig. 3). Screw positions were classified into four grades: grade 0, no perforation, and the screw was completely contained in the pedicle; grade 1, perforation <2 mm (that is, less than half of the screw diameter); grade 2, perforations ≥2 mm but <4 mm; and grade 3, perforation ≥4 mm (complete perforation). A total of 452 CPSs were placed using a preoperative 3D CT-based navigation system (first period of 2007–2012), and a total of 310 CPSs using an intraoperative 3D CT-based navigation system (second period of 2012–2015), so that 762 CPSs were placed in the spine level of C2 to Th3. In the first period of 2007–2012, accuracy of CPS placement was 93.6 % (423 of the 452 screws) in grade 0, 3.1 % (14 of the 452 screws) in grade 1, 2.7 % (12 of the 452 screws) in grade 2, and 0.6 % (3 of the 452 screws) in grade 3. In the second period of 2012–2015, accuracy of CPS placement was 97.1 % (301 of the 310 screws) in grade 0, 2.3 % (7 of the 310 screws) in grade 1, 0.3 % (1 of the 310 screws) in grade 2, and 0.3 % (1 of the 310 screws) in grade 3. Accuracy of CPS placement was also analyzed based on spine level. Screw malposition was found 0.9 % (1 of 114 screws) at C2, 5.5 % (5 of 91 screws) at C3, 5.9 % (8 of 135 screws) at C4, 4.9 % (6 of 122 screws) at C5, 3.3 % (3 of 88 screws) at C6, 10.2 % (10 of 98 screws) at C7, 5.9 % (4 of 68 screws) at T1, 2.9 % (1 of 34 screws) at T2, and 0 % (0 of 12 screws) at T3. The overall rate of screw malposition rate at C2-C6 (where VAs are usually running in the transverse foramen) was 4.1 % (23 of 550 screws). Illustrative case of posterior cervical instrumented fixation using CPS was presented in Figs. 4 and 5

In total, 38 of the 762 screws (5.0 %) were found to perforate the cortex of pedicle in the first and second period, although any neural or vascular complications closely associated with CPS placement were not encountered in the present study. There was no case of vertebral artery injury related to CPS placement. Twenty nine of the 38 screws (76.3 %) were found to perforate laterally, 7 screws (18.4 %) were found to perforate medially, and 2 screws (5.3 %) were found to perforate inferiorly. There were no screws that were found to perforate superiorly. Accuracy of CPS placement was summarized in Table 2.

**Table 2:** Accuracy of CPS placement

Spinal level	No. of CPSs	Grade 0	Grade 1	Grade 2	Grade 3	Grade 1–3 (%)	CPS misplacement			
							Medially	Laterally	Inferiorly	Superiorly
C2	114	113	0	0	1	0.9	1	0	0	0
C3	91	86	4	1	0	5.5	1	4	0	0
C4	135	127	5	3	0	5.9	2	6	0	0
C5	122	116	4	2	0	4.9	1	5	0	0
C6	88	85	2	1	0	3.3	0	2	1	0
C7	98	88	3	5	2	10.2	0	9	1	0
T1	68	64	3	0	1	5.9	0	4	0	0
T2	34	33	1	0	0	2.9	0	1	0	0
T3	12	12	0	0	0	0	0	0	0	0
Total	762	724	22	12	4	5.0	7	29	2	0

*CPS* cervical pedicle screw

## Discussion

In the present study, we focused our attention on the safe CPS placement for posterior cervical instrumented fixation. Careful imaging analysis demonstrated that a total of 38 of the 762 screws (5.0 %) were found to perforate the cortex of pedicle in the first and second period, although any neural or vascular complications closely associated with CPS placement were not encountered. CPS misplacement was more recognized in C7 compared to the other spine level, and the misplacement to lateral direction tended to occur in mid-lower cervical spine. CPS at C7 is usually applied in case of posterior cervical long fusion, and placed at the caudal side of fusion segment. The procedure of CPS placement at C7 level may be easily affected by the inward pressure of well-developed paravertebral muscle layers. The stronger the axial trajectory is kept against the medial pressure from the paravertebral muscles, the easier the axial rotation or sagittal bending of the cervical spine might be, so that screws may be misplaced laterally. The accuracy of CPS placement may be also affected by anatomical complexity of cervical pedicle or navigation error.

In 1991, Panjabi et al. published the first three-dimensional anatomical study of the human cervical vertebrae [21]. The three-dimensional coordinates of various marked points on the surface of the vertebra were measured with a specially designed morphometer instrument. Transverse pedicle diameter increased from 5.1 mm at C3 to 6.6 mm at C7, and sagittal diameter increased from 6.7 to 7.6 mm across the same levels. Karaikovic et al. investigated cervical pedicle morphology using human cadaver specimens via manual and computed tomography measurements and suggested that there were anatomical limitations associated with the use of the pedicle screw in the cervical spine [22, 23]. Chazono et al. evaluated the linear and angular parameters of the cervical spine as well as pedicle dimensions required for safe CPS placement using multiplanar CT reconstruction [24]. They reported that the overall mean pedicle transverse angle (PTA) ranged from 33.6 to 50.2° and that the smallest mean PTA was found at C7 in males (33.4°) and in females (34.1°); the largest mean PTA was found at C4 in males (50.4°) and in females (49.6°) followed by C5, C3, and C6. Further, the pedicle sagittal angle (PSA) varied from 13.7° in the cephalad direction at C3, 5.3° at C4, 1.3° caudally at C5, 3.0° caudally at C6, and 3.3° caudally at C7. The suggested that not only pedicle dimensions but also linear and angular parameters of the cervical vertebral body can be useful data for safe CPS placement. Wang et al. demonstrate their technique of free-hand subaxial CPS placement without using intraoperative navigation devices, and reported clinical results in which C3 showed a higher perforation rate than the other vertebrae [25]. They analyzed the transverse angle of CPS trajectory and perforation of the lateral wall by using postoperative CT scans, and suggested that there are two crucial maneuvers for increasing accuracy of screw placement: identifying the precise entry point and guiding CPS trajectory on axial plane by the resistant of thick medial wall. Shin et al. reported that the pedicle size and angle as well as the shapes of the cervical pedicle are important for safe CPS insertion [26]. Twenty-six human cervical vertebrae from fresh-frozen spines were secured to a thin sectioning apparatus to produce three 0.7-mm-thick pedicle slices along its axis. Radiographs taken of these pedicle slices were scanned, digitized, and traced to facilitate visual comparison. The pedicle slices were found to exhibit substantial variability in composition and shape, not only between individual spines and vertebral levels but also within the pedicle axis. They suggested that the lateral cortex was consistently found to be thinner than the medial cortex in all samples. They reported that pedicle axial slices in the mid-cervical vertebra exhibit a semi-circular or rectangular or triangular shape, with the flat surface directed towards the lateral aspect of each pedicle. As the slices progress antero-posteriorly along the pedicle long axis, the shape of the pedicle slices changes to a more circular conformation. Surprisingly, there are some slices that exhibited an unusual hook shape at their inferior aspects. These anatomical findings must be noted by surgeons attempting CPS placement for posterior cervical instrumented fixation.

The percentage of CPS misplacement varies from 2.5 to 29.1 % in the literature (Table 3) [3, 16, 20, 27–36]. In the present study, 6.4 % of CPS misplacement of grade 1–3 in the first period and 2.9 % in the second period were recognized. Intraoperative image guidance is an important issue to avoid the surgery-related complications. Abumi et al. reported that 45 of 669 inserted screws (6.7 %) were misplaced in his early series [3]. Neo et al. reported that the rate of malposition was 29 % in patients with degenerative conditions [20]. Hojo et al. reported that the overall rate of malposition was 158 of 1065 inserted screws (14.8 %) in their retrospective multicenter study of free-hand insertion technique with fluoroscopy [16]. Even with a preoperative 3D CT-based navigation system, there is always a risk of misplacement of CPS that may result in significant neural or vascular complications. Intervertebral anatomical relationships with the patient in the prone position during surgery might not match the preoperative CT data obtained while the patient was in the supine position. This discrepancy has led to navigation errors. Intraoperative 3D CT-based navigation system may be the solution for navigation errors. It can reduce the discrepancy so that it may provide greater accuracy and feasibility during CPS placement. Ito et al. reported a malpositioning rate of 2.8 % (5 of 176 screws) for CPS placement using a 3D fluoroscopy-based navigation system (Iso-C^3D^C-arm, Siemens Medical Solutions, Erlangen, Germany) [29]. Ishikawa et al. also reported that a 3D fluoroscopy-based navigation system can improve the accuracy of CPS when compared with a conventional insertion technique [27]. Intraoperative 3D CT-based navigation system is the cutting edge of image-guided surgery that includes the acquisition of high-resolution images with 3D data during the surgery and enables surgeons to use them as fully automatic navigation. Although the quality of O-arm images may not be fully comparable to the images of multidetector helical CT, particularly at the cervicothoracic junction which is crucial for CPS, surgeons may not need to rely on the preoperative CT images in which the patient’s position may vary from the surgical position during surgery.

**Table 3:** Review of the literature focusing on accuracy of CPS placement

Author	Year	No. of patients	No. of CPSs	CPS malposition		Intraoperative image guidance
				No.	%	
Abumi et al.	2000	180	667	45	6.7	Fluoroscopy
Kotani et al.	2003	17	78	1	1.2	Preoperative CT-based (Kotani probe)
Neo et al.	2005	18	86	25	29.1	Fluoroscopy
Richter et al.	2005	20	93	8	8.6	Preoperative CT-based
		32	167	5	3	Preoperative CT-based + cannulated screw
Ito et al.	2008	50	176	5	2.8	3D fluoroscopy-based
Yukawa et al.	2009	144	417	59	14.3	Fluoroscopy
Miyamoto et al.	2009	29	130	5	3.8	CT cutout technique
Ishikawa et al.	2010	30	126	34	27	Fluoroscopy
		32	150	28	18.7	3D fluoroscopy-based
Ishikawa et al.	2011	21	108	12	11.1	Intaoperative CT-based
Kawaguchi et al.	2012	11	44	2	4.5	Template system
Tauchi et al.	2013	46	196	24	12.2	Preoperative CT-based
Hojo et al.	2014	283	1065	158	14.8	Fluoroscopy
Uehara et al.	2014	129	579	116	20	Preoperative CT-based
Kaneyama et al.	2015	20	80	2	2.5	Template system
Present study		89	452	29	6.4	Preoperative CT-based
		39	310	9	2.9	Intaoperative CT-based

CPS misplacement of grade 2 and grade 3 is clinically most critical. CPS misplacement of grade 2 and grade 3 in the present study was 3.3 % (15 of the 452 screws) using a preoperative 3D CT-based navigation system, and CPS misplacement of grade 2 and grade 3 was 0.6 % (2 of the 310 screws) using an intraoperative 3D CT-based navigation system. This advancement in image-guided surgery can improve the accuracy of CPS placement to secure the safe surgery. Lateral fluoroscopy in addition to computer navigation was also crucial and used for the accurate guidance of CPS placement, especially in case of high cervical or mid-cervical spine. However, in case of lower cervical or upper thoracic spine, CPS placement was basically guided only by using navigation, not lateral fluoroscopy, because the patient’s shoulder or scapula may disturb the surgeons to see the direction of CPS clearly. Additionally, several technical considerations without navigation system must be discussed. Abumi et al. originally demonstrated his original technique to create a funnel-like-shaped hole at the screw insertion point down to the entrance of the pedicle to secure a wider range of freedom for the screw insertion angle [2, 37]. Yukawa et al. used pedicle axis views generated by fluoroscopy during CPS placement [36]. Miyamoto et al. described a CT cutout technique to navigate the direction of the pedicle [32]. Recently, a patient-specific screw guide template system has been also introduced [30, 38].

## Conclusion

The use of CPS placement for posterior instrumented fixation needs to be carefully determined based on the individual pathological and anatomical condition. A thorough knowledge of surgical anatomy, biomechanics, and technique remains the most essential aspect for successful surgery. Various attempts to avoid misplacement of CPS were performed but have not been completely successful. Therefore, we should continue to attempt to minimize the malposition rate by using a variety of techniques. Image-guided CPS placement has been an important advancement to avoid the surgery-related complications.
